# Hospital Administration

**Published:** 1895-02-02

**Authors:** 


### THE INSTITUTIONAL WORKSHOP.

### HOSPITAL ADMINISTRATION.

[Questions and correspondence, marked "Hospital Administration" on the envelope, addressed to the Editor, are invited for this column.]

### LONDON HOSPITALS AND THE COUNTY COUNCIL.

Lieutenant-Colonel Charles Ford, one of the members of the County Council for the district of North Lambeth, proposed the following resolution at a recent meeting of the County Council, which was, however, rejected by a large majority :—

That it be referred to the General Purposes Committee to consider and report as to the sufficiency or otherwise of the general hospital accommodation for the poor in that part of London south of the Thames; and as to the desirableness of the Council contributing out of the rates to the funds of the London hospitals, the ratepayers to be represented by the nominee of the Council on the governing body of each hospital receiving such financial assistance.

There appears to have been little or no discussion on the point. We thought the matter of sufficient interest to ask Colonel Ford to state the grounds which induced him to move on the matter. In response to our inquiries Colonel Ford writes as follows :—

The London County Council is so near its dissolution that all it can comfortably do is to clear up its business, and its members get ready for the next election.

My motive on the above subject was not, therefore, seriously considered, in my opinion. It goes without saying that South London (one division of which I have the honour to represent) is greatly in want of more general public hospitals. There are, in fact, only two south of the Thames. I hold that public hospitals are as indispensable in great cities as parks and open spaces; as good water; as good house drains; as good poor laws, and the wise administration of such laws. Indeed, I am not sure that the poor law ought not to include good hospital accommodation for the poor.

Why on earth should our hospitals, of all public institutions be "supported by charitable contributions"? Why? Look at the City of Berlin where the hospitals belong to the City Council, and are under its management. Why not in London; the greatest city in the world. If every public general hospital in London was rate-supported, still the special hospitals, *i.e.*, those which treat of special diseases, would absorb much of the contributions of the charitably disposed.

Then, again, are the London hospitals at present well managed—economically managed? Would there not be a great saving all along the line if they were all placed under one central authority?

The constitution of such an authority might be on the lines of the Technical Education Board for London, or on the lines of the Thames Conservancy Board.

The hospitals should certainly be rate-aided, and when London gets those new and those additional sources of income for its County Council, there should not be any great difficulty from a finance point of view in thus aiding our hospitals, especially if a workable system is devised under which each parish was allotted its own hospital, to which, out of local rates, it should be required to contribute. Further, there should be public convalescent homes established in the country for London hospital patients, but with this branch of the subject I need not now deal. Should I be a member of the next County Council, I shall take further action in the matter.

The Berlin Corporation contributes about £70,000 a year to its city hospitals out of the rates. Given new sources of income, and a more just payment to London out of the Exchequer contributions, and I should be prepared to vote, say £150,000 a year from the London County Council funds towards our hospitals.

It will be seen that Colonel Ford has evidently not studied the hospital question, and that he has much to learn in regard to the resources and present position of the voluntary hospitals. It is somewhat amusing to find that Colonel Ford's idea is, as we understand him, to transfer all the great voluntary hospitals to the rates, and to leave the special hospitals a free field to gather in all the contributions they can get from the public, so that they may increase and multiply exceedingly. We should recommend Colonel Ford to get particulars of the Dublin hospitals, which are subsidised at the present time, and to read the report presented to Parliament by the Hospital Commission in Dublin, and also the report of the Dublin Hospital Sunday Fund. We are convinced that if he will do this his views will be considerably modified, especially if he extends his reading by obtaining a report of the investigations into the Eastern Hospital, a rate-supported institution, which reveals the dangers and evils to the system which he at present advocates. In this country rate-supported institutions are the only institutions which are practically shut out from the public eye. It is more difficult to obtain admission to a rate-supported hospital or kindred institution than to any other building of any kind in this country. At the present time the voluntary hospitals, the efficiency of which places them at the head of all medical institutions in the world, owe much of their efficiency to the circumstances that every little matter which occurs within their walls is liable to be made public, and that anyone who is interested in their work may inspect them at all reasonable hours, and so judge their work on its merits. There are many other reasons why the proposal to place the hospitals on the rates must be withstood to the death, but we believe that the one reason which will prevent the great English hospitals from being degraded to this extent is that as at present conducted they offer the maximum of security to the patients and the public.

